# Epiretinal membranes in patients with uveitis: an update on the current state of management

**DOI:** 10.1007/s10792-024-03199-2

**Published:** 2024-06-28

**Authors:** Dimitrios Kalogeropoulos, Andrew John Lotery, Bhaskar Gupta, Stephen Lash, Serafeim Antonakis

**Affiliations:** 1 Southampton Eye Unit, University Hospital Southampton, Tremona Road, Hampshire, Southampton, SO16 6YD UK; 2 Faculty of Medicine, University of Southampton, Southampton, UK

**Keywords:** Uveitis, Inflammation, Cytokines, Epiretinal membrane, Pars plana vitrectomy

## Abstract

**Purpose:**

This review aims to summarize the current knowledge concerning the clinical features, diagnostic work-up, and therapeutic approach of uveitic epiretinal membranes (ERM).

**Methods:**

A thorough investigation of the literature was conducted using the PubMed database. Additionally, a complementary search was carried out on Google Scholar to ensure the inclusion of all relevant items in the collection.

**Results:**

ERM is an abnormal layer at the vitreoretinal interface, resulting from myofibroblastic cell proliferation along the inner surface of the central retina, causing visual impairment. Known by various names, ERM has diverse causes, including idiopathic or secondary factors, with ophthalmic imaging techniques like OCT improving detection. In uveitis, ERM occurrence is common, and surgical intervention involves pars plana vitrectomy with ERM peeling, although debates persist on optimal approaches.

**Conclusions:**

Histopathological studies and OCT advancements improved ERM understanding, revealing a diverse group of diseases without a unified model. Consensus supports surgery for uveitic ERM in progressive cases, but variability requires careful consideration and effective inflammation management. OCT biomarkers, deep learning, and surgical advances may enhance outcomes, and medical interventions and robotics show promise for early ERM intervention.

## Introduction

Epiretinal membrane (ERM) is an abnormal layer that develops at the vitreoretinal interface, defined by the proliferation of myofibroblastic cells associated with extracellular matrix (ECM) on the inner surface of the central retina along the inner limiting membrane (ILM) [1, 2]. Because of its contractile properties and their impact on the underlying retina, it can result in substantial visual impairment, specifically reduced visual acuity (VA) and metamorphopsia. ERM has been referred to by various names, including macular pucker, cellophane maculopathy, primary retinal folds, wrinkling of the inner retinal surface, preretinal macular fibrosis (PMF) or gliosis, and silent central retinal vein obstruction [3]. This retinal disorder can be caused by a wide spectrum of factors and pathophysiological pathways [1, 2]. While the development of ophthalmic imaging techniques, such as optical coherence tomography (OCT), has revolutionized the detection and grading of ERM [4] and other vitreoretinal abnormalities [5], it has not been able to offer additional insights into the histopathological variations, highlighting the heterogeneous nature of this group of diseases [1]. ERMs can be either idiopathic or secondary [1, 2]. Cell proliferation in idiopathic or primary ERM typically occurs after posterior vitreous detachment (PVD) [6], with a prevalence of up to 95%, along with a disruption in the ILM [4]. On the other hand, secondary ERMs occur as a consequence of preexisting ocular pathologies, such as vitreoretinal vascular disorders. Some of the most common causes encompass intraocular inflammation (e.g., uveitis) [7], proliferative diabetic retinopathy (PDR) [8], proliferative vitreoretinopathy (PVR) [9], hypertensive retinopathy, central (CRAO) or branch retinal vein occlusion (BRAO), vitreous haemorrhage, retinal tears, retinal detachment (RD), ocular trauma, photocoagulation, and various retinal surgical procedures [2, 10–14]. In eyes with uveitis, the occurrence of ERM, either with or without cystoid macular edema (CME), is a frequent complication [15–20]. Similar to idiopathic ERM, the preferred surgical approach is pars plana vitrectomy (PPV) with ERM peeling. Nevertheless, there are ongoing debates surrounding various aspects of the surgical approach for uveitic ERM, such as optimal timing, perioperative therapeutic management, and the potential combination of ILM peeling [21]. This review aims to summarize the current knowledge on the management of ERM in patients with uveitis.

## Epidemiology and risk factors

The epidemiology of ERMs initially relied heavily on population-based studies that utilized non-mydriatic retinal photography. Subsequent studies incorporated ocular coherence tomography (OCT) for ERM detection [11, 22]. The Blue Mountains Eye Study (BMES) [24] and the Beaver Dam Eye Study (BDES) [23] were two early large-scale population studies that reported a prevalence of idiopathic ERM (ERM) at 7% [24] and 11.8% [23], respectively. These studies also reported a 5-year cumulative incidence of 5.3% [25] based on color fundus photographs. In the 20-year follow-up of the BDES, spectral domain (SD)-OCT was employed for ERM detection, revealing a prevalence of 34.1% [22], significantly higher than the 11.8% detected through fundus photography [22, 23]. A meta-analysis of 13 population-based studies calculated an overall ERM prevalence of 9.1% [26].

The majority of existing studies on ERMs predominantly concentrate on idiopathic ERM, which is primarily associated with aging and PVD [3]. The incidence of ERM, with or without CME, in patients with uveitis varies from 12.6 to 69% [15–20]. The frequency of ERM according to SUN classification stood at 28.1% in cases of anterior uveitis, 57.0% in intermediate uveitis, and 43.5% in instances of posterior and panuveitis [18]. While ERM is a relatively common complication in uveitic eyes, obtaining data regarding demographics, diagnosis, and disease management can be challenging due to the relative paucity of these patients [21]. Several studies have reported their findings on the evaluation and/or surgical approach of ERMs in uveitic patients [6, 27–33]. However, these results may not accurately represent the true incidence of ERMs resulting from intraocular inflammation.

In patients with uveitis, inflammation may persist or occur in periodic flare-ups layered on top of a baseline of ongoing inflammation. It has been suggested that the resulting vision loss is often a consequence of cumulative damage caused by multiple episodes of inflammation, rather than being attributed to a single initiating event. Therefore, the course of uveitis is characterized by recurrent bouts of inflammation, with each episode contributing to the progressive and permanent deterioration of visual acuity and several complications such as ERMs and CME [34].

The impact of race and ethnicity on the prevalence of ERM has yet to be fully elucidated [4, 12, 35]. However, these variations could potentially stem from differences in study methodologies, such as sampling techniques and photography, or discrepancies in the definition of ERM, particularly when clinical signs are not readily apparent [36]. Ethnic disparities may also be influenced by genetic predisposition, clinical factors, coexisting conditions (e.g., diabetes), prior cataract surgery, and lifestyle factors (e.g., smoking). Furthermore, exposure to unidentified risk factors may play a role. Nevertheless, advancing age remains the most prominent risk factor for all types of ERM [37].

## Histopathology and pathogenetic mechanisms

Notably, uveitic ERMs tend to result in poorer VA outcomes compared to idiopathic ERMs. This disparity may arise from distinct mechanisms of formation or the heightened ocular morbidity associated with uveitis when compared to other diseases [18, 29, 38, 39]. Understanding the underlying pathophysiology of ERMs in patients with uveitis is crucial for the development of innovative therapeutic approaches and effective management strategies. ERMs typically comprise two layers that overlay the ILM. The outermost layer, located atop the ILM, consists of non-cellular ECM proteins containing bundles of extracellular fibrils arranged in a random orientation. Above this layer lies an inner cellular sheet composed of a single or multi-layer of epiretinal cells. The epiretinal cells present in ERMs can originate from either the retinal or extraretinal sources. These cells encompass various types, including glial cells such as Müller cells and astrocytes, retinal pigment epithelium (RPE), fibrocytes, myofibroblasts, fibroastrocytes, laminocytes, hyalocytes, macrophages, and fibroblasts [10, 40, 41]. The outer layer of the ERM’s ECM consists of extracellular fibrils, remnants of the ILM, and residual vitreous fibrils in conditions like vitreoschisis or partial PVD [42]. Additionally, this layer contains proteins such as fibronectin (FN), vitronectin (VTN), and collagen [40–42]. As ERMs progress, the accumulation of myofibroblast-like cells and the deposition of ECM increase, enhancing their contractile properties [42]. ERMs can exhibit vascularization, particularly in eyes with proliferative retinopathies, or they can be avascular [43]. However, secondary ERMs, which develop as a result of other ocular conditions, are generally less aggressive and rarely lead to tractional retinal detachment (TRD) compared to primary ERMs [44]. Idiopathic ERMs are characterized by nonangiogenic fibroglial tissue, whereas ERMs associated with PDR are primarily characterized by neovascularization [45, 46]. ERM represents the most advanced stage of PDR, as it can cause retinal traction or tractional retinal detachment due to the contraction of the ERM [47]. However, the molecular mechanisms underlying the formation of both idiopathic and secondary ERMs are not well understood [45]. It is suspected that glial cell proliferation, which occurs in PDR, plays a crucial role in ERM formation [45, 48]. Fibrocellular proliferation of the ILM and cellular contraction are also features of ERMs. PVD can result in ILM injury, facilitating the movement of glial cells to the retinal surface and providing favorable conditions for fibrocellular proliferation between the vitreous and the retina. An incomplete PVD may contribute to these conditions as well [40, 49, 50].

While there may be clinical similarities, the precise mechanism underlying the development of ERM in uveitis remains uncertain when compared to other disease processes. Nevertheless, histologic and immunohistochemical examinations have successfully distinguished uveitis-associated ERMs from idiopathic ERMs. This distinction is based on the notable presence of abundant inflammatory cells (e.g., lymphocytes, neutrophils, histiocytes, plasma cells, and occasional eosinophils) and the absence of RPE cells within uveitic ERMs [51–54].

Recent studies have emphasized the significance of inflammation and fibrosis in various age-related conditions, including ERM [55]. The examination of inflammatory cytokines in eyes with idiopathic ERM has shown that several cytokines, such as GRO-α, IL-8, and MCP-3, exhibited elevated levels in these patients following uncomplicated cataract surgery [56]. Therefore, similarly, assessing the changes in the specific intraocular cytokine microenvironment in uveitic patients could potentially contribute valuable insights into the pathogenic mechanisms of inflammatory ERM. Apart from that, defining the exact role of cytokines and their alterations in an immune-privileged anatomical site, such as the human eye, is critical in cases of diagnostic uncertainty. This improved diagnostic ability could be crucial in patients suspected of primary vitreoretinal lymphoma [57, 58].

It appears that various inflammatory mediators are involved in the pathogenesis of uveitis and its associated complications, such as ERM. To determine the patterns of these intraocular inflammatory mediators in patients with uveitis, Fukunaga H et al. [59] carried out an analysis of 21 inflammatory cytokines, 7 chemokines, and 5 colony-stimulating/growth factors in vitreous samples from 57 uveitis cases associated with intraocular lymphoma (IOL, n = 13), sarcoidosis (n = 15), acute retinal necrosis (ARN, n = 13), or bacterial endophthalmitis (BE, n = 16). For control purposes, they analyzed samples from eyes with idiopathic ERM. Through heat map analysis they recorded distinct patterns of inflammatory mediators in the vitreous humor of uveitis-afflicted eyes, specific to each disease. More specifically, they found a significant elevation of interferon-α2 in ARN and an increase in interleukin (IL)-6, IL-17A, and granulocyte-colony stimulating factor in BE. In the pairwise comparisons between IOL, ARN, and BE, we discovered that the levels of IL-10 in IOL, RANTES (regulated on activation, normal T cell expressed and secreted) in ARN, and IL-22 in BE were notably higher compared to the other two forms of uveitis. These identified mediators likely play a role in the immunopathology of specific uveitis types and have the potential to serve as valuable biomarkers. Probably, future studies will be able to shed light in the inflammatory mediators associated with uveitic ERM and indicate potential therapeutic targets.

Interestingly, the incidence of drug-induced uveitis has witnessed a notable increase in recent years, primarily attributed to the development of novel biological agents used in the treatment of various types of tumors. Effectively managing these adverse events necessitates a collaborative approach involving both oncologists and ophthalmologists. It is imperative to individualize the management strategy based on a careful assessment of the risk-benefit balance. Mozo Cuadrado et al. [60] highlighted the occurrence of uveitis and subsequent development of an epiretinal membrane during the administration of Dabrafenib, Trametinib, and subsequently Nivolumab for metastatic cutaneous melanoma. This study underlined the significance of diligent follow-up for patients undergoing treatment with these medications.

It appears that a great variety of molecules and mechanisms are involved in the pathogenesis of ERM, but providing a detailed description of these is beyond the scope of this review. For a more in-depth analysis reader is referred to the article of Tsotridou et al. [2].

## Clinical features

ERMs have been associated with a negative impact on the quality of life and can lead to significant visual impairment [36, 37]. These membranes are characterized by various pathological changes that occur at the vitreoretinal junction, presenting with diverse clinical signs [1]. On dilated fundoscopy, ERMs may manifest as translucent, transparent, or pigmented membranes, or even exhibit a glistening light reflection when they are thin [11, 42]. While early-stage ERMs that are thin and translucent tend to be asymptomatic or cause minimal changes in visual acuity, typically not dropping below 20/40, more severe forms characterized by thicker and contractile membranes can result in significant visual acuity loss, sometimes falling below 20/200, and various visual symptoms. [3, 11, 42, 61]. ERMs can be located peripherally or in the macular region, with macular involvement having a greater visual disruptive impact [62]. They can affect the macular and/or perimacular regions, leading to reduced visual acuity, micropsia, metamorphopsia, retinal wrinkling, distortion, blurred vision, and occasionally monocular diplopia [4, 10, 42, 63]. However, the occurrence of absolute scotomas is rare [3]. ERMs can also induce vitreoretinal tractions or even tractional retinal detachment [10] by exerting traction on the underlying retina, particularly when the macula becomes detached due to traction [3, 43]. The symptoms experienced by individuals can vary depending on the duration and severity of the disease [10].

## The role of ophthalmic imaging

**Fig. 1:**
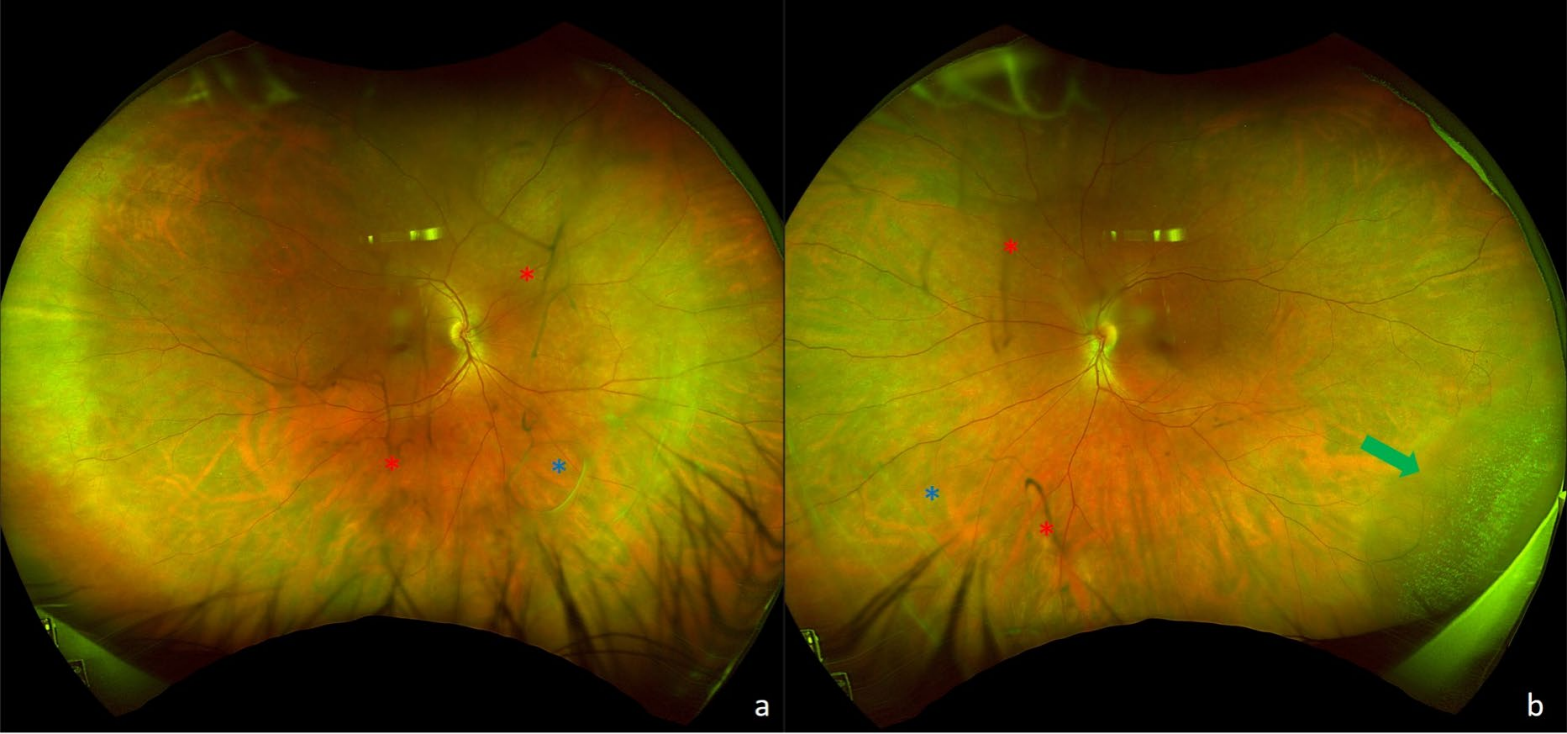
A 42-year-old male with a history of bilateral intermediate uveitis. Optos widefield photography of the right (**a**) and left (**b**) eye illustrates bilateral vitreous debris/opacities (red asterisks) and abnormalities of the vitreoretinal interface (blue asterisks) secondary to the intraocular inflammatory activity. An epiretinal membrane (ERM) is present in both eyes, but it is more prominent in the right eye. An inferotemporal retinoschisis (green arrow) can also be observed in the left eye. No vitritis or other inflammatory features can be seen in these photos. Spectralis SD-OCT (Heidelberg Engineering, Heidelberg, Germany) shows the extent and anatomical features of the ERM; absence of vitreomacular traction, and no evidence of cystoid macular oedema (**c**: right eye, **d**: left eye). SD-OCT, Spectral Domain Optical Coherence Tomography

**Fig. 2:**
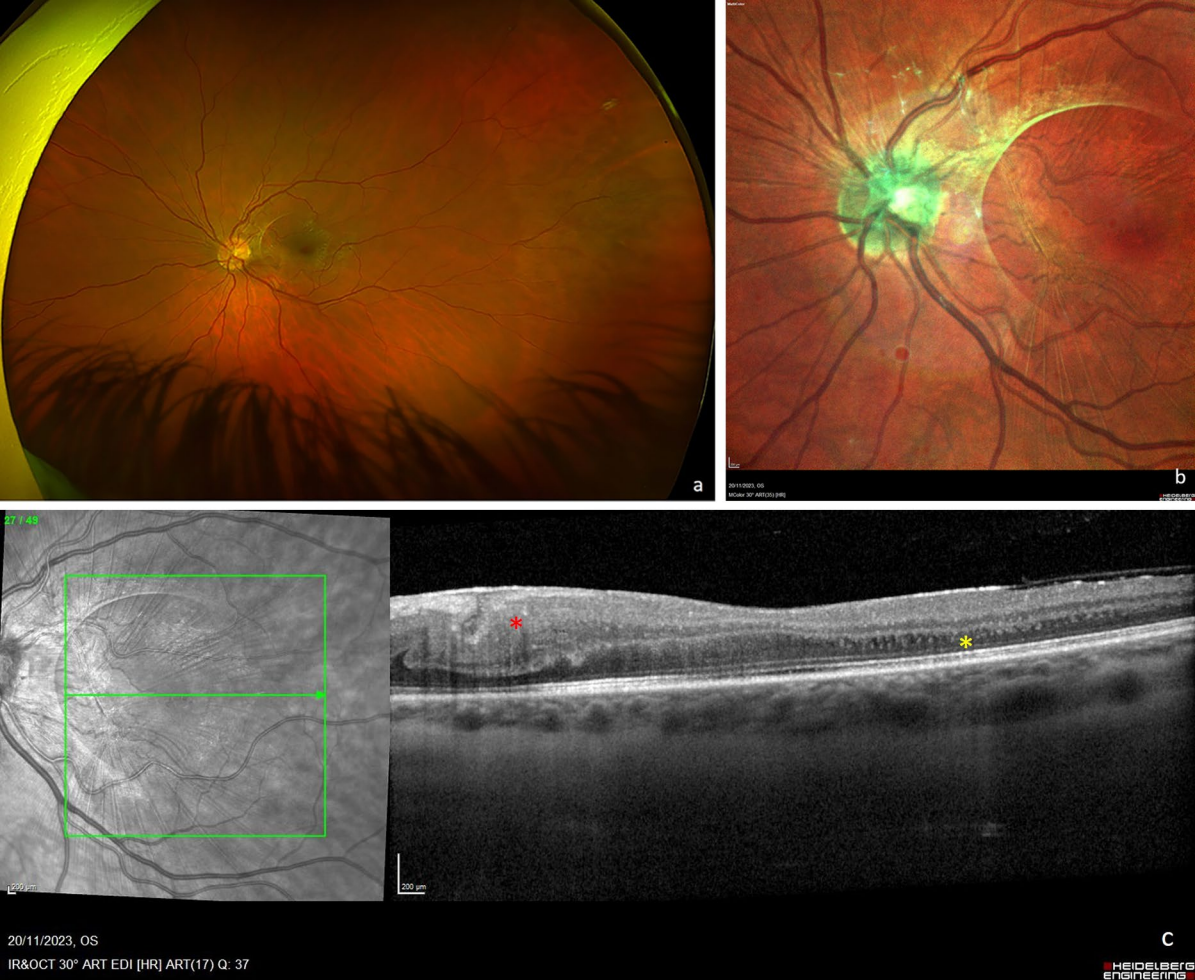
A uveitic epiretinal membrane (ERM) in the left eye of a 30-year-old female as depicted by different imaging modalities. Although Optos widefield photography **a** shows clearly the presence of the ERM, Spectralis MultiColor (Heidelberg Engineering, Heidelberg, Germany) **b** illustrates in detail the extent and tractional features of the ERM. Spectralis SD-OCT (Heidelberg Engineering, Heidelberg, Germany) **c** shows that there is no significant vitreomacular traction. A retinal thickening can be observed nasally (red asterisk). Moreover, a few tiny intraretinal chronic cystic spaces can be seen temporally (yellow asterisk). SD-OCT, Spectral Domain Optical Coherence Tomography

Nowadays, SD-OCT has become an integral part of routine uveitis practice. In addition to its diagnostic value in uveitis, OCT has facilitated the assessment of treatment response and provided valuable insights into visual recovery and prognosis of uveitic conditions [7, 30]** [**Figs. 1 and 2**]**. It is considered the standard diagnostic technique for detecting and monitoring uveitic macular edema, as well as other inflammatory macular disorders such as ERM formation, vitreomacular traction, foveal atrophy, and macular holes of various depths. Moreover, OCT has played a crucial role in unraveling the underlying mechanisms of several posterior uveitic conditions. With the help of SD-OCT, it has become possible to visualize and analyze four distinct lines within the sensory retina. These lines correspond to the external limiting membrane, the photoreceptor inner and outer segment junction, the photoreceptor outer segment and the retina pigment epithelium junction, and the retina pigment epithelium-choriocapillaris complex. Overall, SD-OCT has not only enhanced diagnostic capabilities in uveitis but has also provided valuable information for monitoring treatment progress and predicting visual outcomes. Its application extends to various macular pathologies associated with inflammation, enabling a deeper understanding of the pathophysiology of these conditions [7]. Therefore, SD-OCT is mandated in all uveitic patients, whereas ERM is more common among those with panuveitis and intermediate uveitis [64].

An SD-OCT-based study by Iannetti et al. [6] evaluated retrospectively 41 eyes of 32 patients with uveitic ERM. The final best-corrected VA (BCVA) showed a positive correlation with the male sex (*P* = 0.0055) and the focal pattern of ERM attachment (*P* = 0.031). On the other hand, it had a negative correlation with the disruption of the IS/OS photoreceptor junction (*P* = 0.042). The change in BCVA was positively correlated with the age of ERM onset (*P* = 0.056). However, it showed a negative correlation with IS/OS photoreceptor disruption at the time of ERM diagnosis (*P* = 0.029) and an increase in central subfield thickness (CST) (*P* = 0.95). The final ERM thickness was correlated with the duration of uveitis (*P* = 0.0023) and the duration of ERM (*P* = 1.15e−05). During the follow-up period, ERM thickening was associated with the male sex (*P* = 0.042), posterior uveitis (*P* = 0.036), longer duration of uveitis (*P* = 0.026), and a broad attachment pattern (*P* = 0.052).

A retrospective cohort and cross-sectional study of eighty consecutive eyes with uveitis and SD-OCT-documented ERM showed that VA remains stable if intraocular inflammation and other comorbidities are effectively addressed [65]. Similarly, the same team [32] conducted a longitudinal morphometric analysis of ERM in 6 consecutive eyes with uveitis using SD-OCT. Over a 2 year follow-up period, an increase in ERM thickness in cases of well-controlled intraocular inflammation does not result in vision loss.

The utilization of multimodal imaging is highly beneficial as it offers valuable insights into various abnormalities linked to uveitis. Apart from ERM, this includes the visualization of retinal and choroidal lesions, vasculitis, optic nerve disorders, retinal ischemia, and macular edema. By employing multimodal imaging techniques, a wide spectrum of uveitic manifestations can be effectively displayed and assessed [7, 66].

## Management of intraocular inflammation

Complications of uveitis primarily depend on the severity of inflammation, with acute anterior uveitis accompanied by a reaction of ≥3+ and vitreous haze of ≥3+ being more frequently associated with complications [7]. Other factors contributing to complications include the underlying etiology (e.g., acute retinal necrosis, serpiginous choroiditis, birdshot chorioretinopathy, Adamantiades-Behçet’s disease), recurrence rate, inadequate treatment, and prolonged inflammation. Vision impairment typically arises from recurrent episodes or chronic inflammation, leading to what is known as “cumulative damages”, a term introduced by Quan Dong Nguyen in 2006 [34]. Timely referral to a specialist clinic, the overall duration of the disease, the anatomical location of the inflammation (anterior, intermediate, posterior, or panuveitis), and the frequency of uveitis episodes significantly impact the final disease outcome [67, 68]. Developing diagnostic and therapeutic algorithms that offer comprehensive guidelines for approaching patients with uveitis enables clinicians to initiate timely and appropriate treatment, ultimately reducing the severity and frequency of cumulative damages [7].

## Medical management

Currently, there are no specific medical treatments available for ERM. However, macular edema associated with certain secondary causes of ERM, such as diabetic retinopathy, retinal vein occlusion, and uveitis, may respond to intravitreal administration of anti-VEGF (vascular endothelial growth factor) agents, steroids, or non-steroidal agents. Research in the field of vitreopharmacolysis explores the use of biological enzymes to dissolve ERMs. Although intravitreal ocriplasmin has been studied for its efficacy in vitreomacular traction (VMT) associated with ERM, its effectiveness in resolving the membrane itself remains uncertain [69]. Phase III clinical trials evaluating the use of ocriplasmin in subjects with VMT included a subset of patients with ERM, but the impact of ocriplasmin on the membrane was unclear due to the small sample size [70].

## Surgical management

**Fig. 3:**
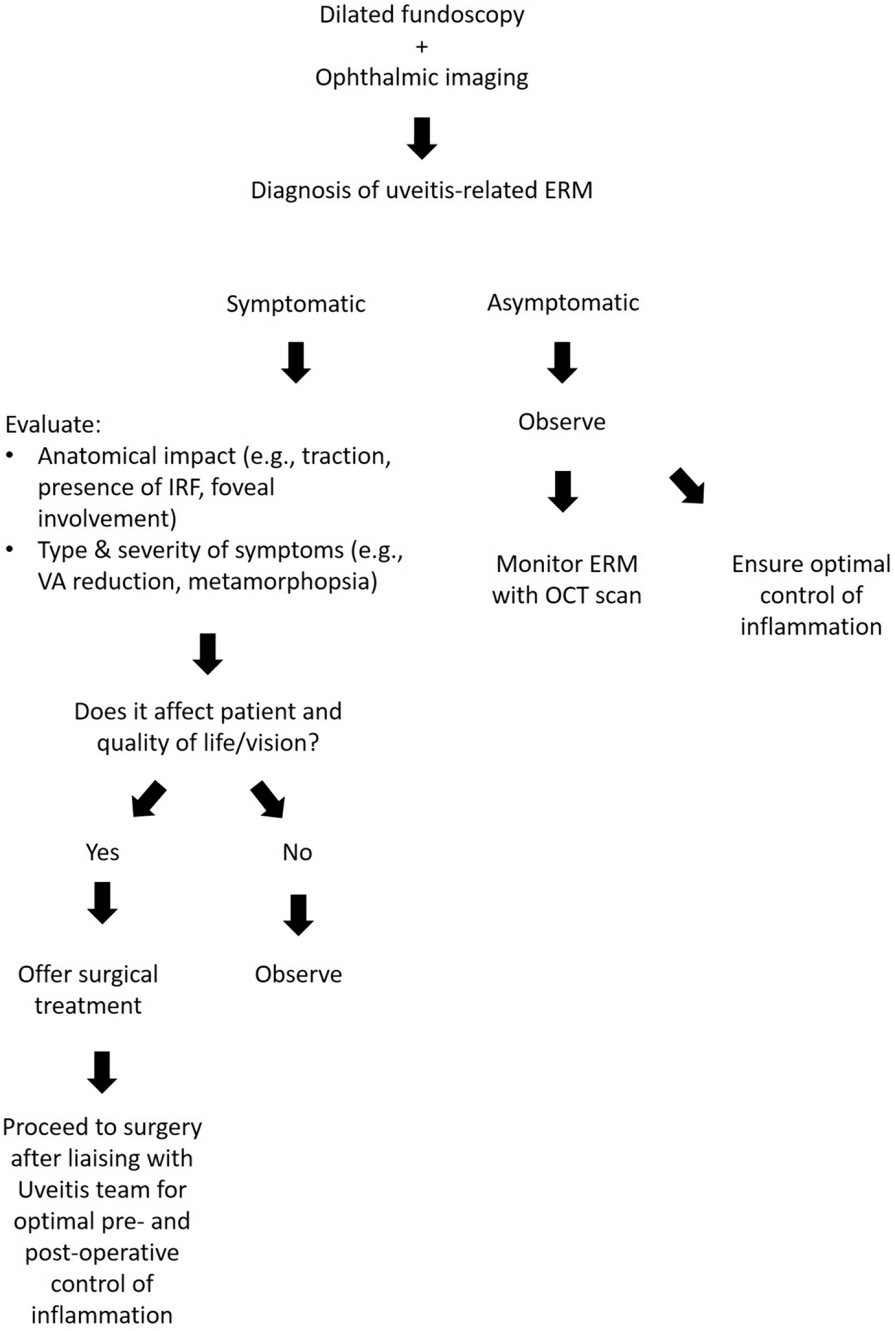
Suggested protocol for the management of epiretinal membranes in uveitic patients. ERM, epiretinal membrane; IRF, intraretinal fluid; OCT, Optical Coherence Tomography, VA, visual acuity

In the realm of surgical management **[**Fig. 3**]** for complications associated with uveitis (e.g., CME, ERM, macular hole, and RD), several factors have significantly contributed to enhancing surgical outcomes. These factors encompass advancements in surgical techniques, utilization of smaller incisions, improved therapeutic approaches, enhanced perioperative care, increased surgical experience, and the development of novel types of intraocular lenses (with acrylic hydrophobes being regarded as the most assessable option) [71]. A comprehensive clinical examination plays a crucial role in formulating a preoperative surgical plan. It is emphasized that the eye should be in a state of rest for a minimum of 3 months before undergoing ophthalmic surgery [7]. Preoperative prophylaxis involving corticosteroids is vital in minimizing the risk of macular edema and uveitis recurrence. Additionally, antimicrobial prophylaxis can effectively reduce reactivation risks in eyes affected by infectious uveitis. In cases where inflammation is poorly controlled or in patients under the age of two, it is advisable to delay intraocular lens implantation. Vigilant monitoring of patients is essential to detect any signs of disease recurrence, inflammation, elevated intraocular pressure, hypotension, or other complications. Through meticulous patient selection, refined surgical techniques, and optimization of perioperative and postoperative care, favorable visual outcomes can be achieved in individuals with uveitis [72–74].

Although the spontaneous resolution of inflammatory ERM has been reported [75], the only treatment for ERM is surgical removal, and advancements in vitrectomy systems have facilitated less invasive procedures. Nevertheless, standardized criteria for ERM surgery are currently lacking, and the decision to perform surgery relies on the patient’s subjective symptoms [76]. The consensus among authors is that surgical intervention for ERM in uveitis is appropriate for eyes experiencing progressive structural and functional deterioration. However, due to potential outcome variability in certain cases (when compared to idiopathic ERM), such surgery should only be recommended once intraocular inflammation is effectively managed and conservative approaches have proven ineffective [77]. PPV has become increasingly utilized in uveitic cases for both diagnostic and therapeutic purposes [31, 77–79]. The introduction of microincision vitrectomy surgery (MIVS) has expanded the indications for PPV in uveitis due to its advantages over the conventional 20G vitrectomy, including shorter surgical duration, reduced patient discomfort, minimized conjunctival scarring, and decreased complications. This has led to a rise in the popularity of MIVS in uveitis, as it enables faster postoperative recovery in terms of visual improvement, inflammation reduction, and reduced reliance on systemic corticosteroids [80]. The safety and efficacy of MIVS can be attributed to the development of advanced vitrectomy techniques incorporating improved cutters, lighting systems, and auxiliary instruments/tools. Consequently, PPV utilizing MIVS has emerged as a safe and valuable alternative for diagnosing challenging uveitic cases, aiding in early detection and better management of inflammatory diseases, even in the presence of severe and active inflammation, which was previously considered a relative contraindication for vitreous surgery [81]. However, when performing PPV for therapeutic indications and uveitis-related complications, it is advisable to achieve optimal control of inflammation to optimize outcomes. The increasing number of reports documenting the successful use of MIVS in uveitis has led to its broader acceptance among uveitis specialists.

Cristescu et al. [21] evaluated retrospectively the surgical outcomes of 29 eyes of 29 consecutive uveitic patients with ERM. In particular, this study investigated the anatomical and functional outcome of PPV and ERM peeling and secondarily the effect of ILM peeling on these eyes. The average duration of follow-up in the study was 32 months, with a standard deviation of 22 months. According to their findings, at the 6 month follow-up, there was a significant improvement in mean central retinal thickness (CRT), with values decreasing from 456 microns (with a standard deviation of 99 microns) to 353 microns (with a standard deviation of 86 microns) (*p* < 0.001). Additionally, mean BCVA showed improvement, with values improving from 0.73 logMAR (with a standard deviation of 0.3 logMAR) to 0.49 logMAR (with a standard deviation of 0.36 logMAR) (*p* < 0.001). Only one patient (3%) experienced a worsening of vision. The incidence of concurrent CME decreased from 19 eyes (66%) at the beginning of the study to eight eyes (28%) at the final follow-up (*p* = 0.003). When comparing eyes where ILM peeling was performed in addition to ERM peeling alone, there was no significant difference in BCVA improvement or CRT reduction. A small proportion of the eyes (six, or 21%) experienced worsening of uveitis that required additional medications, while the majority of patients either continued the same treatment (52%) or required less treatment (28%) (*p* = 0.673). Furthermore, the findings from this study suggest that in cases where there is no activation of uveitis necessitating the use of additional medications, no adjustments should be made to the postoperative anti-inflammatory/immunosuppressive therapy.

A retrospective interventional case series of 16 patients that underwent PPV with ERM peel showed that after 6 months, VA improved in 31.25% of eyes, remained stable in 31.25%, and deteriorated in 37.5%. Factors contributing to the reduced visual acuity following the surgery included severe preexisting macular pathology and untreated cataracts [29]. Performing PPV with ERM peel in uveitic eyes has the potential to enhance or maintain visual acuity, particularly in cases involving macular traction. However, when traction is absent, the outcomes can vary and become unpredictable. It is crucial to prioritize aggressive control of inflammation to prevent the formation of ERM [29].

These findings come in agreement with the study of Rao et al. [82] who evaluated 15 patients (17 eyes) who had PPV with ERM and ILM peeling. The outcomes were favorable with significant improvement in mean central foveal thickness (517 vs. 371 microns; *P* = 0.01) and macular cube volume (12.1 vs. 9.4 mm3; *P* = 0.01) 6 months after the vitrectomy. No ERM recurrences were noted and there was a trend toward improved mean postoperative VA at 6 months (0.8 [± 0.6] vs. 0.6 [± 0.6] logarithm of the minimum angle of resolution units; *P* = 0.36). Similar to previous studies, the authors underlined the importance of pre- and post-operative control of inflammation.

A more recent study by Coassin et al. [28] investigated the potential benefit of PPV with ERM and ILM peeling in patients with post-uveitic ERM. A total of 26 eyes, with no signs of intraocular inflammation for a minimum of 3 months following the discontinuation of immunosuppressive therapy, were operated. The results demonstrated a significant improvement in VA, with a shift from 20/80 to 20/40. Out of the total number of eyes, 20 (77%) experienced an increase in vision, 4 (15%) maintained stable vision, and 2 (8%) reported a decrease. Furthermore, in 14 eyes (54%), the BCVA improved by at least 2 ETDRS lines. Upon conducting a contingency analysis, no statistical difference was found among the various types of uveitis (*p* = 0.46). Additionally, the mean central foveal thickness showed improvement in the postoperative period (428 ± 104 vs. 328 ± 130 microns; *p* = 0.017). Consequently, patients with uveitic ERM can derive significant benefits from vitrectomy with membranectomy. However, it is crucial to perform the surgery once the intraocular inflammation has subsided.

Other authors have investigated the efficacy of PPV in specific uveitic entities such as sarcoidosis [83] or toxoplasmosis [84], suggesting that vitrectomy is a safe and effective procedure in these categories of patients.

Yap et al. [85] 216 eyes, accounting for 4% of a total of 5450 eyes with uveitis, were found to have an ERM. The most prevalent diagnoses were idiopathic uveitis (28.7%), followed by sarcoidosis (13.4%), HLA B27-related uveitis (9.6%), and toxoplasmosis (9.6%). Risk factors for ERM development included age, intermediate uveitis, posterior uveitis, and having an ERM in the fellow eye. Conversely, anterior uveitis and alternating disease were protective. Median VA remained stable at 20/40 from diagnosis of ERM to final follow-up. ERM progression was observed in 7.9% of cases, while ERM peel was performed in 20.4%. Following surgery, median VA improved from 20/60 at baseline to 20/40 at 12 months, with 60.5% experiencing improved visual acuity.

Although PPV has evolved significantly since its inception almost five decades ago, the quality of evidence in the literature concerning its application for uveitis has not shown similar improvement. While certain structural complications of uveitis may exhibit positive responses to surgery, the visual outcomes still entail some level of uncertainty. Conversely, diagnostic vitrectomy with appropriate ancillary testing remains an important method for evaluating and diagnosing uveitis in a considerable number of patients [77]. Table 1 provides a summary detailing the surgical outcomes in patients with uveitis undergoing intervention for epiretinal membranes.

**Table 1:** Surgical outcomes in patients with uveitis undergoing intervention for epiretinal membranes

Study	Number of patients	Surgical technique	Visual acuity results	Outcome measures	Main findings
Cristescu et al. [21]	29	PPV with ERM peel (with/without ILM peel)	Improved: mean BCVA from 0.73 to 0.49 logMAR; 3% worsened vision	BCVA, CRT, CME, uveitis activity	Significant improvement in CRT and BCVA; CME reduced; majority had stable or reduced uveitis activity; ILM peel did not significantly affect outcomes
Coassin et al., [28]	26	PPV with ERM and ILM peel	Improved: mean BCVA from 0.60 to 0.30 logMAR	BCVA, CMT, inflammation	Visual acuity improved significantly; majority had improved or stable vision; reduced CMT; intraocular inflammation controlled preoperatively
Tanawade et al. [29]	16	PPV with ERM peel (with/without ILM peel)	Improved or stabilized: mean BCVA at baseline was 0.50, at 12 months after surgery was 0.30 logMAR	BCVA, anatomical outcomes	Visual acuity improved or stabilized in most; severe pre-existing macular pathology and unoperated cataract were causes of worsened vision; aggressive control of inflammation important to prevent ERM formation
El Faouri et al. [31]	27	PPV without macular intervention	Improved: significant improvement in BCVA	BCVA, intraocular inflammation	Significant improvement in BCVA; reduction in inflammation; decreased need for systemic steroids and second-line immunosuppressives; resolution of macular edema in most cases
Rao et al. [82]	17	PPV with ERM and ILM peel	Improved: mean BCVA at baseline was 0.8 logMAR, at 6 months after surgery was 0.6 logMAR	BCVA, CFT, macular volume	Significant improvement in CFT and macular volume; trend toward improved visual acuity; no ERM recurrences; some required additional immunomodulatory therapy; minimal postoperative complications
Kiryu et al. [83]	11	PPV	Improved: majority achieved visual acuity of 0.30 logMAR or better at final visit	BCVA, CME, complications	Majority gained Snellen visual acuity; cystoid macular edema resolved in most; postoperative complications included cataract formation, glaucoma, and membrane recurrence; subsequent surgeries needed in some cases
Miranda et al. [84]	14	PPV	Improved: mean preoperative BCVA was 1.00 logMAR, mean postoperative BCVA was 0.50 logMAR	BCVA, complications	Significant improvement in visual acuity; no intraoperative complications; most frequent postoperative complications were posterior capsule opacification and cataract; no disease recurrences
Yap et. al. [85]	216 (44 surgeries)	PPV with ERM peel	Improved: median visual acuity at baseline was 0.30 logMAR, maintained at final follow-up	BCVA, ERM progression	Median visual acuity improved post-surgery; majority maintained long-term vision; low rates of ERM progression; intermediate and posterior uveitis and fellow eye involvement are strong risk factors for ERM development

BCVA, Best-Corrected Visual Acuity; CFT, Central Foveal Thickness; CME, Cystoid Macular Edema; CRT, Central Retinal Thickness; ERM, Epiretinal Membrane; ILM, Internal Limiting Membrane; OCT, Optical Coherence Tomography; PPV, Pars Plana Vitrectomy

## Epiretinal membranes in children

The occurrence of epiretinal membranes in children is uncommon, and they are commonly attributed to traumatic, idiopathic, or uveitic causes. Surgical intervention in such cases has been reported to typically lead to favorable outcomes for patients [86]. However, the current literature lacks data concerning the management of inflammatory ERMs in the pediatric population.

Generally, it must be underlined that managing uveitis in pediatric patients presents significant challenges due to their unique characteristics [87]. While uveitis is less prevalent in children compared to adults, it can be more complex due to the often-asymptomatic nature of the condition or the limited ability of children to communicate their symptoms. Pediatric uveitis can manifest as chronic, persistent, recurrent, or resistant to conventional treatment. Among children, anterior uveitis is the most common type, although the prevalence of intermediate, posterior, and panuveitis varies among ethnic groups and geographical regions. Although many cases of pediatric uveitis have no identifiable cause (idiopathic), it is essential to consider potential underlying etiologies. Uveitis in children can be associated with systemic inflammatory disorders, infections, or masquerade syndromes. Timely recognition and differentiation of these etiologies are crucial to prevent further complications and loss of vision. The therapeutic approach to pediatric uveitis depends on the underlying cause and the unique characteristics of each patient. Regular monitoring is vital to detect complications and adverse effects of treatments at an early stage. A multidisciplinary approach is essential to provide comprehensive care to young patients and their families, ensuring an improved quality of life.

## Future perspectives

As in idiopathic ERM [88], the development of further OCT biomarkers would provide further insights into the evaluation of patients with ERM attributed to uveitis [7]. The integration of artificial intelligence (AI) and machine learning into healthcare has opened up new possibilities for enhancing screening and diagnosis in the field of uveitis. Various applications of AI in uveitis research, specifically focusing on its role in supporting diagnosis, detecting findings, screening patients, and standardizing uveitis nomenclature have been described so far [89]. It has been suggested that deep learning algorithms could contribute to evaluating the degree of visual impairment in individuals with ERM using OCT images. In their study, Hsia et al. [90] developed two convolutional neural network models (ResNet-50 and ResNet-18). Both models d a training accuracy of 100%. However, when tested, the accuracy was 70% for ResNet-18 and 80% for ResNet-50. Utilizing the t-distributed stochastic neighbor-embedding approach, it was observed that the deeper ResNet-50 structure exhibited superior discrimination in identifying OCT characteristics associated with visual impairment compared to the shallower ResNet-18 structure. The heat maps generated from the models revealed that the significant features contributing to visual impairment were predominantly located within the inner retinal layers of the fovea and parafoveal regions. Thus, it has been suggested that alterations in the inner retinal layers have a more significant influence on visual acuity compared to changes in the outer retinal layers [90]. However, it is important to note that the overall performance of AI models in this context is currently limited. This can be attributed to factors such as inadequate datasets, a lack of validation studies, and a scarcity of publicly available data and codes. Despite these limitations, the potential of AI to aid in the diagnosis and detection of ocular manifestations of uveitis is rather promising. Nonetheless, further research and the inclusion of large, representative datasets are imperative to ensure the generalizability and fairness of AI applications in this field [89].

## Conclusion

Histopathological studies and advancements in retinal imaging, particularly OCT, have significantly enhanced our understanding of the pathophysiology of ERMs. However, the challenge of developing a single pathogenic model that can explain all clinical scenarios implies the existence of a heterogeneous group of diseases. The consensus among authors is that surgical intervention for epiretinal membrane (ERM) in uveitis is appropriate for eyes experiencing progressive structural and functional deterioration. However, due to potential outcome variability in certain cases (when compared to idiopathic ERM), such surgery should only be recommended once intraocular inflammation is effectively managed and conservative approaches have proven ineffective. The identification of prognostic biomarkers on OCT, along with the integration of deep learning techniques and advancements in surgical procedures, holds the promise of further improving visual outcomes. Additionally, medical interventions and the application of robotics offer the potential for earlier intervention in ERM cases.
